# The network architecture of value learning

**DOI:** 10.1162/netn_a_00021

**Published:** 2018-06-01

**Authors:** Marcelo G. Mattar, Sharon L. Thompson-Schill, Danielle S. Bassett

**Affiliations:** Department of Psychology, University of Pennsylvania, Philadelphia, PA, USA; Department of Bioengineering, University of Pennsylvania, Philadelphia, PA, USA; Department of Psychology, University of Pennsylvania, Philadelphia, PA, USA; Department of Bioengineering, University of Pennsylvania, Philadelphia, PA, USA; Department of Electrical and Systems Engineering, University of Pennsylvania, Philadelphia, PA, USA

**Keywords:** Functional connectivity, Cognitive systems, Brain networks, Behavioral adaptability, Valuation system, Reinforcement learning

## Abstract

Value guides behavior. With knowledge of stimulus values and action consequences, behaviors that maximize expected reward can be selected. Prior work has identified several brain structures critical for representing both stimuli and their values. Yet, it remains unclear how these structures interact with one another and with other regions of the brain to support the dynamic acquisition of value-related knowledge. Here, we use a network neuroscience approach to examine how BOLD functional networks change as 20 healthy human subjects learn the values of novel visual stimuli over the course of four consecutive days. We show that connections between regions of the visual, frontal, and cingulate cortices become stronger as learning progresses, with some of these changes being specific to the type of feedback received during learning. These results demonstrate that functional networks dynamically track behavioral improvement in value judgments, and that interactions between network communities form predictive biomarkers of learning.

## Author Summary

Rational human behavior is the pursuit of actions that maximize expected reward. These rewards can be understood as stimulus-value contingencies, learned by experience throughout our lives. Various structures have been recognized to participate in these learning processes. Yet, an understanding of how these structures interact with one another and with other brain regions remains vastly unexplored. Here, we propose a novel analytical framework utilizing and extending techniques from the dynamic network neuroscience to ask “How do our brains change when we learn values?” We find that interactions between sensory and fronto-cingulate structures grow stronger as learning progresses, bringing together several isolated findings in the cognitive neuroscience of value-based behavior and extending our understanding of human learning in general.

## INTRODUCTION

The behavior of a human is fundamentally driven by their existing notions of value (Simon, 1955). From a vast repertoire of possible actions, humans tend to choose ones that either have been reinforced through prior rewards, or have the potential to bring reward in the future (Montague, King-Casas, & Cohen, 2006; Shizgal, 1997). The concept of value is foundational to decision-making, allowing for diverse alternatives to be placed on a common scale, thereby facilitating choices that maximize expected reward. While a notion of value is intrinsic to many stimuli (e.g., a red apple appears more valuable than a brown apple), in many cases it must be learned through experience (e.g., coffee is more valuable than expected given its appearance). Such conceptual representations of value can be acquired through trial and error (good vs. bad), as well as through learning of declarative information (Delgado & Dickerson, 2012; Packard & Knowlton, 2002; Squire, 1992).

The representation of the value of objects requires the engagement of systems that represent object information and systems that represent value. Visual objects, and their form in particular, are represented throughout the occipital and temporal lobes, occupying part of what is known as the visual system (Felleman & Van Essen, 1991; Grill-Spector & Malach, 2004). Stimulus values, on the other hand, are represented primarily in subcortical and medial prefrontal areas, in a collection of structures referred to as the valuation system (Bartra, McGuire, & Kable, 2013). These include primarily regions of the basal ganglia, anterior cingulate, ventro-medial prefrontal, and orbito-frontal cortices (Bartra et al., 2013). Notably, identifying a stimulus and retrieving its value requires the concerted engagement of both systems.

Numerous studies have attempted to elucidate the specific functions of each region in these systems using clever task designs and sophisticated methodological approaches (Bartra et al., 2013; Cohen, Heller, & Ranganath, 2005; de Beeck et al., 2008; Grill-Spector & Malach, 2004; Grill-Spector & Weiner, 2014; Vassena, Krebs, Silvetti, Fias, & Verguts, 2014). Indeed, the success of these studies is evident by the sheer number of compartmentalized structures identified along with their associated functions. In the valuation system, for example, basal ganglia structures respond in proportion to reward prediction errors (Abler, Walter, Erk, Kammerer, & Spitzer, 2006; O’Doherty et al., 2004; Packard & Knowlton, 2002), a crucial signal in feedback-based learning, while frontal regions of the valuation system tend to respond in proportion to the actual values of stimuli (Bartra et al., 2013; O’Doherty, 2004; Vassena et al., 2014), a signal important for value-based decision-making. However, a fundamental gap in our knowledge lies in delineating how the different cortical and subcortical areas composing the valuation and visual systems interact with one another and with other regions of the brain to allow effective behavioral choices built on the computations of and comparisons between stimulus values. Knowledge of these patterns of interaction is an essential step in moving from a compartmentalized or modular view of brain function, towards a more integrative and dynamic notion of how different regions cooperate to subserve behaviors as complex as perception and decision-making.

Here we address this gap by taking an explicit network neuroscience perspective. This novel analytical framework has encountered great success in characterizing how learning modulates the patterns of statistical dependencies between regional activities, bridging and relating descriptions at the microscale with emerging architectures at the mesoscale (Bassett et al., 2013; Bassett, Yang, Wymbs, & Grafton, 2015; Mattar, Betzel, & Bassett, 2016). In a cohort of 20 healthy adult human subjects, we examine how the pattern of functional interactions between brain regions changes during the learning of monetary values of novel visual stimuli. With over 1,500 trials completed across four consecutive days of practice, participants learned the monetary values of 12 rendered three-dimensional shapes through a feedback consisting either of the value of the shape selected (10 subjects) or whether the selected shape was the most valuable from the pair (10 subjects).

We hypothesized that learning accompanies gradual changes in the architecture of functional networks, with progressively increased integration between visual and valuation systems to allow proficient task performance. We further hypothesized that the recruitment of network regions and modules varies depending on the type of feedback received in the task, with an increased engagement of basal ganglia structures for participants learning by trial and error and an increased engagement of visual structures for participants learning declarative information. Finally, we hypothesized that this effect is strongest in the early stages when learning is more pronounced and a full representation of the stimulus values is incomplete.

Our results not only confirm these hypotheses, but they also suggest that functional networks can almost perfectly distinguish whether a novel subject has already learned the stimulus values, and can significantly classify the type of feedback received in the learning protocol. These findings demonstrate that behavioral improvements in value judgment are represented in patterns of functional connectivity that change in a characteristic manner predictive of learning. Moreover, our study offers a set of novel analytical approaches applicable to the study of human learning specifically and dynamic networks more broadly.

## RESULTS

### Experimental Paradigm

**Figure 1.**
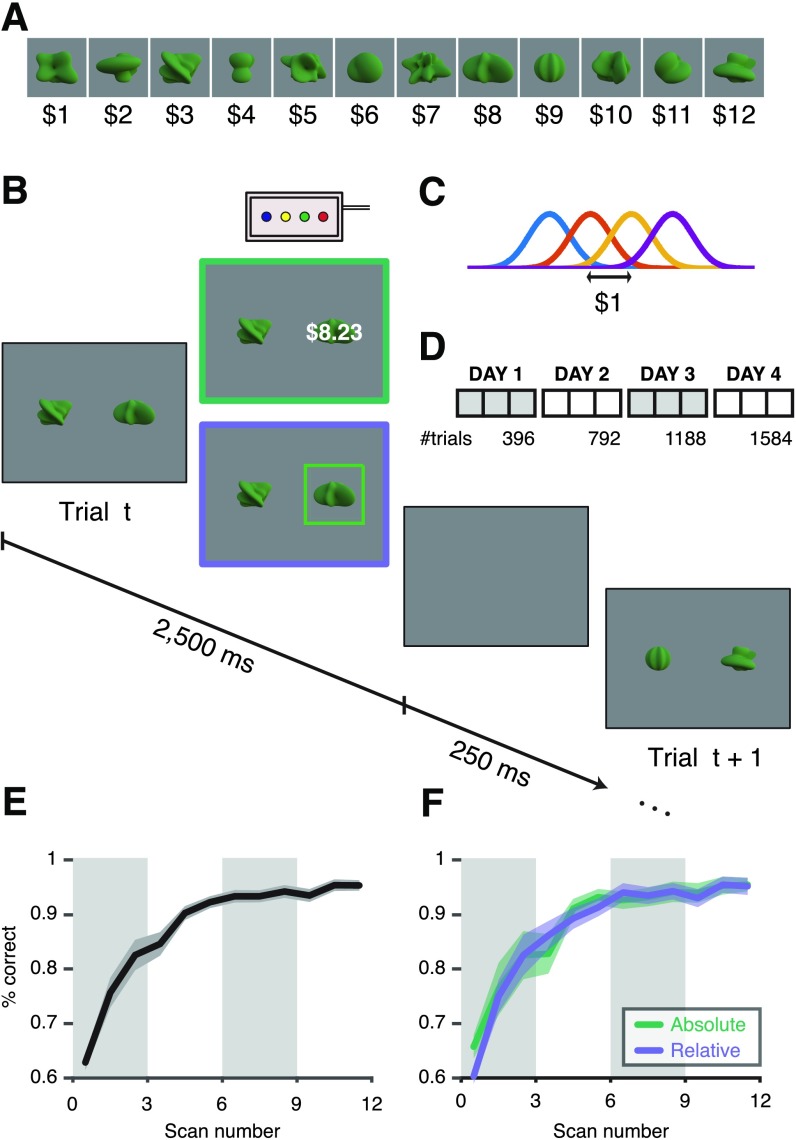
Experimental paradigm and behavioral results. (A) Stimulus set and corresponding values. Twelve abstract shapes were computer generated, and an integer value between $1 and $12 was assigned to each (Figure S2, Mattar et al., 2018). This value remained constant over the four days of training. (B) Task paradigm. Participants were presented with two shapes side by side on the screen and asked to choose the shape with the higher monetary value. Once a selection was made, either the value of the shape selected (absolute feedback) or the correctness of the selection (relative feedback) was provided as feedback. Each trial lasted 2,750 ms (250 ms interstimulus interval). (C) On each trial, the empirical value of each shape was drawn from a Gaussian distribution with fixed mean (i.e., the true value), as described in panel (A), and standard deviation of $0.50. (D) The experiment was conducted over four consecutive days, with three experimental scans (396 trials) on each day, for a total of 1,584 trials. (E) Participants’ accuracy in selecting the shape with higher *expected* value improved steadily over the course of the experiment, increasing from chance level in the first few trials to approximately 95% in the final few trials (*N* = 16). (F) Task accuracy followed a similar profile for all participants in both the absolute feedback (*N* = 8) and the relative feedback (*N* = 8) groups.

Twenty healthy adult human subjects learned the monetary value of 12 novel visual stimuli over the course of four consecutive days (Figure 1A). On each trial of the experiment, participants selected which of two shapes simultaneously present on the screen had the highest value, after which they received feedback based on their response. Participants were randomly assigned to two groups, determining the type of feedback that they would receive. Ten participants received relative feedback: a green (red) square surrounding the selected shape, signaling whether their response was correct or incorrect but not indicating the specific shape values. The other ten participants received absolute feedback: the value of the selected shape was presented to the participant, but not the value of the nonselected shape, nor information about whether the selection was correct (Figure 1B). Although each shape had a true value, the empirical value used for each trial was drawn from a Gaussian distribution with a fixed mean (i.e., true value; Figures 1A and 1C) and with a standard deviation of $0.50. We arrived at this latter choice by performing preliminary behavioral studies to identify a standard deviation value that led to quantitatively similar learning rates in both the relative and the absolute feedback groups.

We collected blood oxygen level dependent (BOLD) functional MRI data from each participant as they performed the task. A total of 12 scan runs over four days were completed by each person (three scans per session), totaling 1,584 trials (Figure 1D). The average accuracy in selecting the shape with the highest mean value at each trial gradually improved over the course of the experiment, increasing from approximately 50% (chance) in the first few trials to approximately 95% in the final few trials. This behavioral improvement was consistent across participants and between feedback groups (Figures 1E and 1F). Two participants from each group were excluded on the basis of head motion and task performance (Figure S1 in the Supplementary Information, Mattar, Thompson-Schill, & Bassett, 2018), with the other 16 subjects contributing data for the main analyses described in this paper.

### Evolution of Functional Networks Throughout Learning

**Figure 2.**
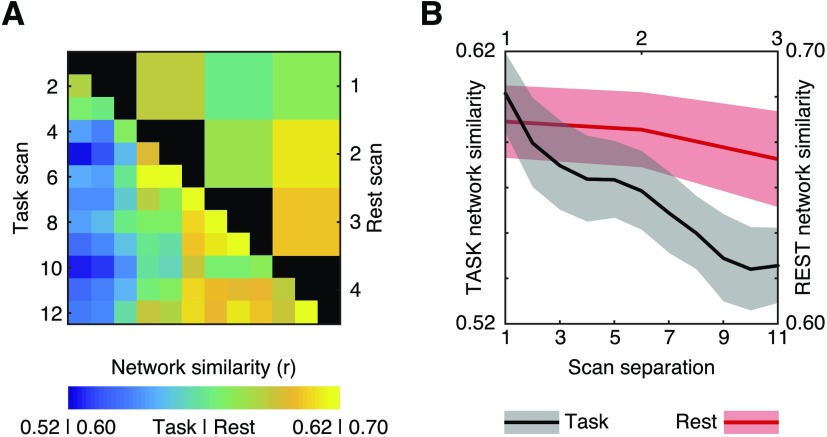
Functional networks underlying task execution evolve slowly over the course of learning. (A) Lower diagonal: Network similarity (Pearson correlation coefficient) between functional connectivity matrices corresponding to each pair of task scans. Upper diagonal: Network similarity (Pearson correlation coefficient) between functional connectivity matrices corresponding to each pair of rest scans, conducted over the same period of time as the task scans (*N* = 16). (B) Average network similarity as a function of temporal separation between corresponding scans. Black line: task scans; red line: resting-state scans (*N* = 16).

Using this value learning task, we first tested whether global changes in functional connectivity occurred concurrently with changes in behavior (Medaglia, Lynall, & Bassett, 2015). We created functional networks (or graphs) for each participant by subdividing their gray matter volume into *N* = 112 cortical and subcortical areas (nodes) and calculating the statistical dependency between the BOLD activity time courses from each pair of nodes (edges; E. T. Bullmore & Bassett, 2011). We defined one such functional network for each of the 12 scans, and we represented that network as an *N* × *N* weighted adjacency matrix. Then, for each pair of scans, we calculated the Pearson correlation coefficient between their associated pair of matrices, intuitively measuring the interscan similarity in the pattern of statistical dependencies between regional BOLD time series. We observed that functional networks were more similar to each other when the corresponding scans were close to one another in time than when they were far from one another in time (Figure 2A). Interestingly, functional networks tended to evolve most from the first to the second day (Scans 1–3 and 4–6), which was also the period that saw the greatest improvement in accuracy (Figure 1E).

We summarized these results by calculating the average functional network similarity (Pearson correlation between adjacency matrices) as a function of scan separation (Figure 2B). A repeated-measures analysis of variance indicated that average network similarity significantly decreased as scan separation increased, *F*(10, 150) = 11.43, *p* < 0.001. As a critical null model, we considered the baseline resting-state scans acquired prior to each task session, in order to rule out potential effects at the session level that were unspecific to learning. A repeated-measures analysis of variance did not yield significant results, suggesting that average functional network similarity at rest did not significantly decrease as rest scan separation increased, *F*(2, 30) = 0.70, *p* = 0.51. Similar results were obtained when separately considering the two feedback groups (absolute: *F*(10, 70) = 3.46, *p* = 0.0010 for task vs. *F*(2, 14) = 0.33, *p* = 0.73 for rest; relative: *F*(10, 70) = 10.51, *p* < 0.001 for task vs. *F*(2, 14) = 0.48, *p* = 0.63 for rest; Figure S3, Mattar et al., 2018). Similar results were also obtained when considering the average functional networks for each day as opposed to each scan, yielding an equal number of time points for the task and rest data (*F*(2, 30) = 7.51, *p* = 0.0023; Figure S3, Mattar et al., 2018), or with global signal removed (Figure S8, Mattar et al., 2018). These results indicate that functional networks evolve steadily during task execution, suggesting their sensitivity to the learning of value information. Importantly, a homogeneous change in functional connectivity in the entire network cannot account for these results (e.g., an overall increase or decrease in connectivity), given that a correlation measure discounts a mean offset. Therefore, the pattern of edge weights changes throughout learning, with distinct connections changing in different ways. We thus turned our attention to changes at a finer scale to effectively characterize the reconfiguration in network architecture associated with learning.

### Relationship Between Behavioral and Network Changes

**Figure 3.**
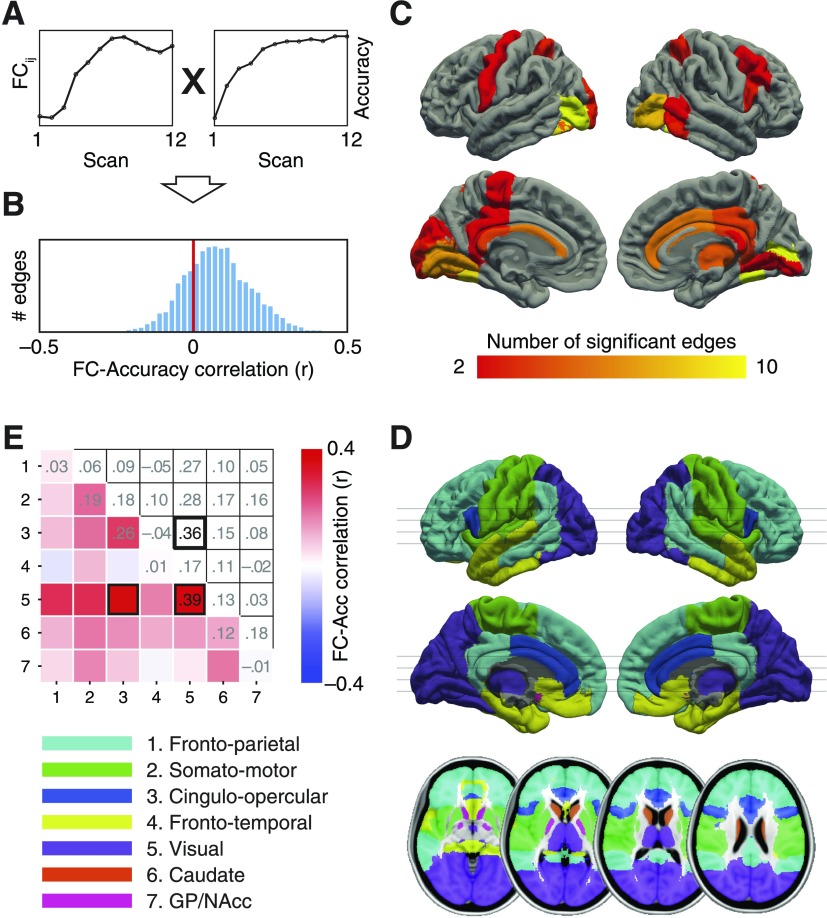
Changes in functional connectivity track changes in task accuracy. (A) Approach for identifying network dynamics associated with behavior. Left inset: The strength of functional connectivity for an example edge connecting nodes *i* and *j*. Right inset: Task accuracy for the same participant. (B) Histogram of Pearson correlation coefficients between edge weight and task accuracy, combining data from all subjects (*N* = 16). Edge weights are, on average, positively correlated with task accuracy, indicating that edge weights tend to increase as learning progresses. (C) Number of edges departing from each node whose variation over time correlated significantly with changes in behavior (*N* = 16). Significance assessed by contrasting the average (Fisher-Z transformed) Pearson correlation coefficient between task accuracy and edge weight, across participants, with a null distribution generated by randomly permuting the order of the scans (Bonferroni corrected at *α* = 0.05). Connections associated with learning primarily involved regions in the visual cortex, but to a lesser degree also regions in cingulate, somato-motor, and dorso-lateral frontal areas. (D) Community structure. Colors represent sets of regions that displayed coherent activity with one another during task execution, across all participants and task sessions. Color code is displayed in the legend in the bottom left of the figure. Subcortical structures are displayed in representative axial slices, shown at the bottom. (E) Correlation between average edge weight within or between communities and task accuracy. Notice the overall trend for positive correlation values, similar to the data presented in panel B. Communities whose interactions were significantly correlated with task accuracy are highlighted in the matrix, with significance assessed by contrasting the average (Fisher-Z transformed) Pearson correlation coefficient between task accuracy and edge weight, across participants, with a null distribution generated by randomly permuting the order of the scans (Bonferroni corrected at *α* = 0.05). Community order is displayed in the legend in the bottom left of the figure.

Having established that functional networks in general evolve steadily over the course of learning, we next turned to an examination of functional changes at the scales of nodes and edges. To quantify the degree to which variation in edge weight over time was associated with learning, we calculated the Pearson correlation coefficient between each participant’s task accuracy and the strength of functional connectivity at each edge in the network, across scans (Figure 3A). We observed a tendency for correlation values to be positive (*M* = 0.079, *SD* = 0.11), suggesting that functional connectivity on average increases as task accuracy increases (Figure 3B). Relatedly, we confirmed that, across subjects, the global network strength was significantly correlated with behavior (*M* = 0.21 ± 0.077 (SEM), one-sample *t* test on Fisher normalized correlation values: *t*(15) = 2.73, *p* = 0.015). These results suggest that behavioral improvements in this task are associated with a global increase in the coherence of BOLD activity.

To rule out the possibility that the increase in global network strength was caused by a changing pattern of motion in the scanner across sessions, we calculated the Spearman rank-order correlation between motion and global network strength across sessions. We found that motion was not significantly correlated with either absolute displacement (RMS displacement relative to a single reference volume; *M* = 0.11 ± 0.10 (SEM), one-sample *t* test on Fisher normalized correlation values: *t*(15) = 1.19, *p* = 0.25) or average relative displacement (RMS displacement relative to the preceding volume; *M* = 0.17 ± 0.10 (SEM), one-sample *t* test on Fisher normalized correlation values: *t*(15) = 1.69, *p* = 0.11).

Despite an overall tendency for functional connectivity values to increase over the course of learning, this profile was not present in all edges of the network (Figure 3B). Thus, we wished to distinguish the parts of the network that were related to behavioral changes from those that did not change or from those that changed in a manner unrelated to behavior. Using a nonparametric permutation-based approach, we compared the mean correlation coefficient between task accuracy and edge weight, across participants, with a null distribution generated by randomly permuting the order of the scans. We then applied a Bonferroni correction for multiple comparisons (112 × 111/2 = 6,216 tests) and counted the number of edges departing from each node whose changes across time were significantly correlated with improvements in task accuracy. We observed that regions of the visual cortex—in particular around the calcarine sulcus, inferior lateral occipital cortex, and posterior fusiform—included the most edges whose weights tracked learning. Following these strongest hubs of behaviorally linked connections were additional regions in cingulate, somato-motor, and dorso-lateral frontal cortices (Figure 3C).

While these results provide information about focal regions from which important edges emanate, they do not address the question of which edges specifically change strength in concert with task accuracy. To examine this finer-scale structure while maintaining interpretability, we categorized edges grouped by the corresponding cognitive systems recruited by value learning. We used a data-driven approach built on a network-based clustering method to uncover these cognitive systems or functional modules. Specifically, we identified groups of brain regions that displayed coherent activity with one another during task execution, forming network communities. Using this approach, we obtained subject-specific communities for every trial block, and obtained a representative consensus partition by identifying the groups of nodes that were consistently assigned to the same community across participants and across time. The consensus partition divides the brain into seven distinct communities: a fronto-parietal community spanning regions of the dorso-lateral, ventro-lateral, and ventro-medial frontal cortices, posterior cingulate, and inferior parietal lobe; a somato-motor community comprised of regions in the precentral and postcentral gyri and sulci; a cingulo-opercular community covering the anterior cingulate and frontal operculum; a fronto-temporal community spanning medial temporal areas, the superior and inferior temporal gyri, and medial orbito-frontal cortex; a visual community composed of the occipital, posterior parietal, and inferior temporal cortices and thalamus; and two subcortical communities: one formed by bilateral caudate, and one formed by the nucleus accumbens and globus pallidus (Figure 3D).

We then used this community structure—largely in agreement with known divisions of the visual and value networks—to summarize groups of network connections that robustly changed with learning. First, we calculated the average edge weight within each community or between each pair of communities, and then we calculated the correlation between these module-level estimates of functional interactions and task accuracy (Figure 3E). As expected from the observed increase in connectivity values over the course of learning, these correlation values were generally positive. To assess the significance of these effects, we compared the average (Fisher-Z transformed) correlation value, across subjects, with a null distribution of 10,000 correlation values obtained by permuting the order of the scans uniformly at random. Two community-level interactions showed significant correlations with task accuracy (Bonferroni corrected at *α* = 0.05): as task accuracy increased, connection strength similarly increased (a) within the visual community (adjusted *p* value: *p* = 0.0028); and (b) between visual and cingulo-opercular communities (adjusted *p* value: *p* = 0.011). Similar results were obtained when analyzing absolute and relative feedback groups separately (Figure S4, Mattar et al., 2018). Together, these results suggest that interactions between visual and cingulo-opercular networks change in accordance with task accuracy.

### Predicting a Person’s Learning Stage From Their Functional Connectivity Pattern

**Figure 4.**
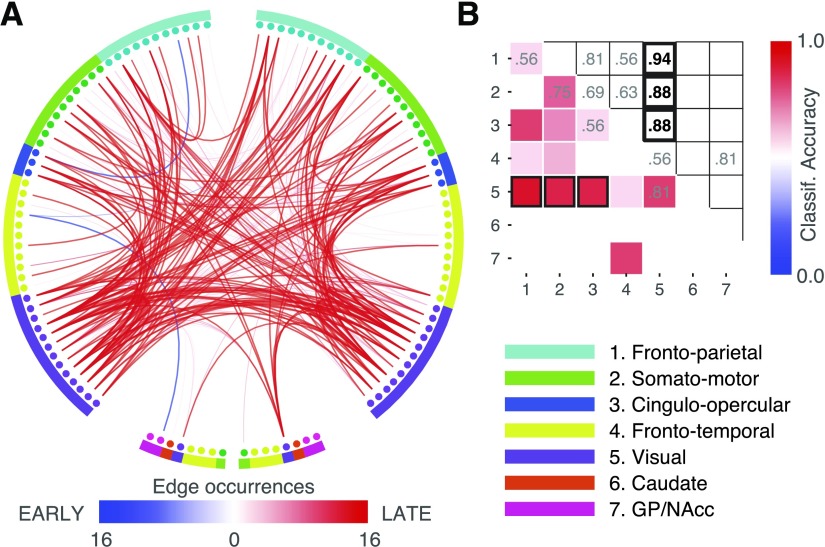
Functional connectivity between network modules predicts a person’s learning stage. (A) Using a cross-validation procedure (*N* = 16), the subset of edges where a one-sample *t* test yielded a test statistic with *p* value lower than 0.001 was selected as a *predictive network*. The figure shows the number of cross-validation folds in which each edge was identified as part of the predictive network. Edges whose strength correlated positively with task accuracy are displayed in red. Edges whose strength correlated negatively with task accuracy are displayed in blue. (B) The same procedure repeated separately for each pair of communities (*N* = 16). Cell colors and numbers represent classification accuracy in labeling held-out data as coming from scans early or late in the learning process. Communities whose interactions classified held-out data significantly (Bonferroni corrected at *α* = 0.05) are highlighted. Community order is displayed in the bottom right.

We next turned to the stricter test of out-of-sample prediction testing whether snapshots of an unseen participant’s network could be correctly classified as coming from early versus late stages in the learning process. We used a leave-one-out cross-validation procedure to select predictive network edges from each set of *n* − 1 subjects, and we compared their strengths in the left-out, independent participant’s data. For each cross-validation fold, predictive network edges were those in which a one-sample *t* test across subjects demonstrated a significant correlation with behavior at *p* < 0.001. The number of edges present in the predictive networks ranged from 180 to 341 (*M* = 222.6, *SD* = 36.6). We then calculated the average strength in this network for the first three scans (Day 1) and for the last three scans (Day 4) of the left-out participant, and we classified the dataset with lower (higher) strength as early (late). Using this approach, we were able to correctly classify 15 out of the 16 participants’ data (accuracy: 93.75% vs. chance: 50%; one-tailed binomial test: *p* < 0.001). The edges that appeared most frequently in the predictive network linked the visual community with itself (purple) and with the fronto-parietal (cyan), cingulo-opercular (blue), and somato-motor (green) communities (Figure 4A).

For a baseline comparison, using a predictive network composed of all (6,216) possible edges we were able to correctly classify data from 12 out of the 16 participants (accuracy: 75% vs. chance: 50%; one-tailed binomial test: *p* = 0.038). The performance of this classifier is a consequence of the overall increase in the average edge strength of the network and not due to the exact pattern of edge strengths. In a supplementary set of analyses, we removed the average network strength at each scan and repeated the classification procedure, forcing the predictive networks to incorporate only edges whose variation with respect to other edges (and not with respect to a baseline) predicted learning session. Using this alternative cross-validation procedure, the number of edges present in the predictive networks ranged from 105 to 154 (*M* = 132.5, *SD* = 13.7) and classification was correct in all 16 participants (accuracy: 100% vs. chance: 50%; one-tailed binomial test: *p* < 0.001; Figure S5, Mattar et al., 2018). The edges that appeared most frequently in the predictive network linked the visual community with the fronto-parietal (cyan), cingulo-opercular (blue), and somato-motor (green) communities, suggesting that these edges can uniquely predict learning stage above and beyond the overall increase in the average edge strength of the network between the first and last scan days. These results confirm that the unique pattern of edge strengths is a robust predictor of a participant’s learning stage.

To gain insight into the specific modules that enable this classification, we repeated these analyses separately for each pair of communities, selecting each time the edges connecting the two communities for which a one-sample *t* test across subjects demonstrated a significant correlation with behavior at *p* < 0.001 (Figure 4B). Using a Bonferroni correction for multiple comparisons (*α* = 0.05), the data from the left-out participant was significantly classified above chance (50%) when the predictive network was composed of edges connecting (a) visual and fronto-parietal modules (accuracy: 93.75%; one-tailed binomial test, adjusted *p* value: *p* = 0.0054); (b) visual and somato-motor modules (accuracy: 87.50%; one-tailed binomial test, adjusted *p* value: *p* = 0.044); and (c) visual and cingulo-opercular modules (accuracy: 87.50%; one-tailed binomial test, adjusted *p* value: *p* = 0.044). The same pattern of results was obtained after removing average network strength (Figure S5, Mattar et al., 2018), demonstrating that connectivity between these modules can predict learning stage above and beyond the overall increase in the average edge strength of the network between the first and last scan day. Together, these results confirm that interactions between visual and fronto-cingulate regions not only track behavioral improvements, but can also be used to significantly determine the amount of training that an unseen person has completed.

### Predicting Feedback Condition From Functional Connectivity

The results presented above characterize network features that change in concert with learning of value, broadly defined. Yet, information about stimulus value can be acquired in different ways, each involving potentially different brain structures (Delgado & Dickerson, 2012). How might network recruitment and plasticity vary with the type of information used for learning? We addressed this question by comparing the functional network architecture during learning between the two feedback groups.

The feedback manipulation introduced in the task design constrains the specific information available for the decision process while maintaining visual stimuli, participants’ behaviors, and the overall task structure identical across conditions. The required mental operations, in turn, are expected to differ between groups despite equivalent visual inputs and motor outputs. Specifically, participants in the absolute feedback group are required to retrieve and compare stimulus-specific value information, while participants in the relative feedback group retrieve pairwise, relative relationships between stimuli based on previously reinforced choices. This is expected particularly early in the learning process, since after extensive training groups may converge to a state where both absolute and relative value information are available for the decision process. We thus hypothesized that task performance and learning may recruit different brain structures to the core value judgment network, particularly early in the learning process. In particular, we hypothesized a relatively greater involvement of basal ganglia structures in the relative feedback group because learning was driven by reinforcement. To make concrete network predictions, we expected to observe a greater coupling of basal ganglia structures to input (sensory) and output (motor) areas in participants of the relative feedback group, with participants of the absolute feedback group displaying stronger direct coupling between input (visual) and output (motor) structures.

A direct comparison of the group networks yielded no significant edges when corrected for multiple comparisons at a significance level of *α* = 0.05. Yet, we observed a trend for the overall network strength to be higher in the absolute feedback group than in the relative feedback group (*p* < 0.059). In order to identify specific network components that differ between conditions, we again proceeded with a data-driven classification procedure, identifying a different predictive network at each cross-validation fold and testing whether held-out data could be correctly labeled according to the type of feedback received by the participant. Specifically, we calculated the average functional connectivity for each participant in the first three scans (Day 1). We then conducted a leave-two-out cross-validation, forming a training set of all participants except for one participant in each group, and conducting a two-sample *t* test across subjects for each network edge. A predictive network for the absolute (relative) feedback group was defined as the set of edges with 1% largest (smallest) *t* statistic, and the strength of the predictive network calculated for the two held-out participants.

**Figure 5.**
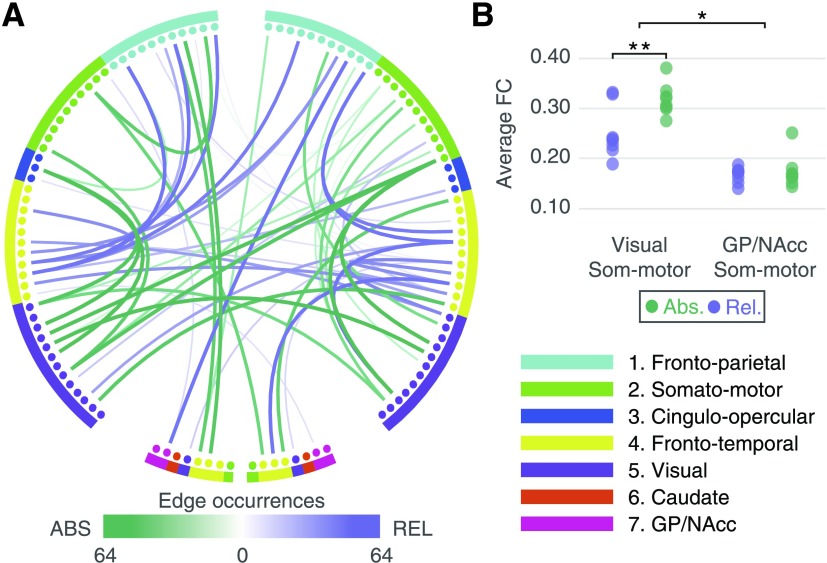
Functional connectivity between network modules predicts feedback condition. (A) Using a cross-validation procedure, the subset of edges with lowest and highest *t* statistics in a two-sample *t* test between absolute and relative feedback were selected as the predictive network for the absolute and relative feedback groups, respectively (*N* = 8 per feedback condition). The figure shows the number of times each edge was part of the corresponding predictive networks (green: absolute feedback; purple: relative feedback). (B) Average functional connectivity between somato-motor and visual, and between somato-motor and GP/NAcc modules displayed separately for each feedback group (*N* = 8 per feedback condition). We observed a significant interaction, with the somato-motor module connecting more strongly to the visual module in the absolute feedback group and to basal ganglia structures in the relative feedback group.

We then asked, for each of the 64 cross-validation folds, whether the strength of this predictive network was sufficient to correctly classify the feedback group of the two held-out participants. Using this procedure, we were able to label the held-out data in 51 of the 64 cross-validation folds, corresponding to 79.69% accuracy (chance: 50%; one-tailed binomial test: *p* < 0.0001). To further assess the significance of this result, we performed a nonparametric permutation test in which the edges composing the predictive networks were selected uniformly at random. This procedure yielded a null distribution of accuracy values centered at chance level (*M* = 0.50, *SD* = 0.062). The observed classification accuracy in the true data (79.69%) was the largest among all 10,000 permutations (*p* < 0.0001). The edges that appeared most frequently in the predictive network for absolute feedback linked the visual community with the somato-motor, fronto-parietal, and cingulo-opercular communities. In contrast, the edges that appeared most frequently in the predictive network for *relative feedback* linked fronto-temporal areas to fronto-parietal and basal ganglia structures—in particular, the nucleus accumbens (Figure 5A). Similar results were obtained when subtracting the mean connectivity from each adjacency matrix in order to account for possible differences in overall network strength between conditions (accuracy: 83.81%), as well as when using a support vector machine to classify feedback condition (accuracy: 80.49%; Figure S6, Mattar et al., 2018). In supplemental analyses at the community level, we observed that feedback could be significantly classified based on interactions involving somato-motor, fronto-temporal, and caudate modules (Figure S6, Mattar et al., 2018).

Next, we examined the consistency of these effects across learning stages. We observed that classification accuracy decreased considerably when data from the final three scans (Day 4) were used to define the predictive network and to classify held-out data (accuracy: 68.75%, albeit still significantly greater than expected in the null distribution, as defined by a nonparametric permutation test: *p* = 0.0017). These results suggest that the networks recruited for the two feedback conditions are distinct in the early stages of training, but become more similar to one another as subjects progressed in learning. We wished to confirm this hypothesis by calculating the similarity between the networks for the two feedback conditions on the first three scans (Day 1) and on the final three scans (Day 4). We observed that the similarity between networks of different type increased from *r* = 0.59 on Day 1 to *r* = 0.65 on Day 4 (*t*(14) = 6.82, *p* < 0.0001). We also observed that the average similarity between the absolute feedback networks for different subjects increased from *r* = 0.68 on Day 1 to *r* = 0.73 on Day 4, and that the average similarity between the relative feedback networks for different subjects increased from *r* = 0.61 on Day 1 to *r* = 0.67 on Day 4. Together, these results confirm that the networks recruited for the two feedback conditions are more distinct in early stages of training than in the later phases of the experiment.

Finally, we explored whether the recruitment of systems hypothesized to be involved in each feedback condition would exhibit a double dissociation. We tested our network predictions by calculating the average strength of connectivity between somato-motor and visual modules, and between somato-motor and basal ganglia (globus pallidus and nucleus accumbens). We observed a significant interaction (two-way ANOVA interaction: *F*(1, 28) = 8.69, *p* = 0.022), with connectivity between somato-motor and visual modules being stronger for the absolute feedback group (two-sample *t* test on Fisher normalized correlation values: *t*(14) = 3.14, *p* = 0.0073), and connectivity between the somato-motor and basal ganglia modules being not significantly different between groups (two-sample *t* test on Fisher normalized correlation values: *t*(14) = 0.51, *p* = 0.62; Figure 5B). The interaction and differences were not significant on Day 4 (two-way ANOVA interaction: *F*(1, 28) = 2.13, *p* = 0.88; two-sample *t* tests: *t*(14) = 0.86, *p* = 0.40, *t*(14) = 1.82, *p* = 0.09; Figure S6c, Mattar et al., 2018). Once again, similar results were obtained after subtracting the mean connectivity from each adjacency matrix (two-way ANOVA interaction: *F*(1,28) = 10.8, *p* = 0.0027; Figure S6, Mattar et al., 2018), which controls for overall differences in network strength between groups. Together, these results indicate a differential recruitment of brain structures for the two feedback conditions, especially early on, with output areas (e.g., somato-motor) being more connected to visual areas or to basal ganglia structures in absolute and relative feedback conditions, respectively.

## DISCUSSION

In this study, we investigate the network-level neural markers of human value learning. Using neuroimaging data collected from a cohort of 20 healthy adults as they learned the monetary value of 12 novel visual stimuli, we demonstrate that functional connectivity—patterns of statistical dependencies between activity in different brain regions—varies in a manner that directly tracks changes in behavior. As accuracy increases, functional connectivity within areas of the visual system and between visual and fronto-cingulate regions also increases. Importantly, these patterns of reconfiguration can be used to predict the learning stage of a single subject, and also to predict which type of learning feedback a subject has been exposed to. Collectively, our results provide strong evidence for the notion that functional brain networks are sensitive to behavioral improvements and task conditions, and offer novel insights into the cognitive neuroscience of value learning.

### Task-Based Network Architecture

Our study begins with a demonstration that functional networks defined during task execution are sensitive to learning, whereas resting-state networks are not (Figure 2A). While the network architectures of rest and task states certainly display some commonalities (Cole, Bassett, Power, Braver, & Petersen, 2014), the present work provides evidence for their differences, complementing a growing set of studies particularly in the context of learning (Bassett et al., 2013, 2015). Indeed, the community structure observed in our study differs from that observed in the resting state (Power et al., 2011), being characterized by modules for visual perception, value comparison, decision-making, and motor responses, as well as a module in which the fronto-parietal and default mode networks are strongly intertwined (Fornito, Harrison, Zalesky, & Simons, 2012).

Notably, the cingulo-opercular network, thought to subserve goal-directed behavior through the stable maintenance of task sets (Dosenbach et al., 2007), played an important role in the present task. This is likely a consequence of it being predominantly composed of the anterior cingulate, an area implicated in a variety of functions such as error detection, conflict monitoring, reward-based learning, and decision-making (Botvinick, Cohen, & Carter, 2004; Bush, Luu, & Posner, 2000; Kennerley, Walton, Behrens, Buckley, & Rushworth, 2006). Indeed, this diversity of functions has been discussed in depth in recent reviews (Holroyd & Yeung, 2012; Shenhav, Botvinick, & Cohen, 2013). While our data are unable to tease apart the specific contribution of the anterior cingulate cortex and the cingulo-opercular network more generally, we speculate that the stable maintenance of a task set involving visual information for reward-based learning is broadly in line with our findings of increased connectivity between this network and visual areas.

Perhaps surprisingly, the network including the medial orbito-frontal cortex (fronto-temporal) did not exhibit the same pattern of changes with learning, despite its hypothesized role in representing the expected rewards of actions. Evidence from neuropsychology, however, seems to indicate that learning decisions based on value feedback does not require the orbito-frontal cortex, relying instead on dorso-medial prefrontal areas (Fellows, 2011)—a finding that our results corroborate.

Finally, the separation of subcortical structures in the basal ganglia into isolated communities (caudate and nucleus accumbens with globus pallidus) is not characteristic of resting-state dynamics, and indicates that these structures have a time course of neural responses that are distinct from other communities, potentially because of signals related to prediction errors (O’Doherty, Dayan, Friston, Critchley, & Dolan, 2003).

### Integration of Visual and Valuation Systems

Models and theory aside, it is important to couch our network-level observations within the cognitive neuroscience of value. Our task paradigm requires participants to process incoming visual information, recognize the stimulus pair, and retrieve the relevant value information, before making a choice and entering a response. With the feedback received, the stored values of the recently observed stimuli are updated so that future choices are optimized for accuracy. Conceptually speaking, both at the time of retrieval and at the time of updating, visual and value-related information must be combined, requiring an intricate interplay between the visual system (Van Essen, Anderson, & Felleman, 1992) and the valuation system (Bartra et al., 2013). Our results directly confirm these predictions by demonstrating that visual areas, and in particular their interactions with areas of the cingulo-opercular module, are strongly modulated by learning, becoming increasingly connected as task accuracy increases (Figure 3E). The increased connectivity between these two modules means that their activity becomes increasingly synchronized with learning. A plausible explanation for this finding is that activity in visual regions becomes increasingly modulated by value—for example, responding more strongly when currently attended stimuli have higher values—similarly to what is observed in regions of the valuation system (Bartra et al., 2013). Indeed, it has recently been observed that visual responses after value learning become modulated by value similarity (Persichetti, Aguirre, & Thompson-Schill, 2015). While our results do not explicitly test for the emergence of multivariate representations that are sensitive to value similarity, they are in direct agreement with this possibility.

### Effect of Feedback

Even in a task as simple as a two-alternative forced choice, the selection of the alternative with highest value may require widely different processes depending on the information previously encoded and currently available. In our paradigm, subjects in the relative feedback group learned ordinal information while those in the absolute feedback group learned cardinal information, and so the content learned differed between conditions. While most of the research on feedback-based learning has been successfully formalized within a reinforcement learning framework, where a positive (or negative) prediction error signal reinforces (weakens) specific stimulus-response associations, this framework cannot account for all forms of learning. In particular, learning of declarative information (in our task also received through feedback), linking a stimulus with a monetary value, is likely to require a different strategy given the lack of a clear reinforcement signal (Packard & Knowlton, 2002; Squire, 1992). These two different learning strategies are thought to recruit distinct memory systems, with basal ganglia regions mediating learning through trial and error (right vs. wrong), and more posterior regions (in particular the medial-temporal lobe) mediating declarative learning (Delgado & Dickerson, 2012; Packard & Knowlton, 2002; Squire, 1992).

Recent evidence suggests that the relationship between these two systems is cooperative rather than competitive (Cincotta & Seger, 2007; Dickerson, Li, & Delgado, 2011; Voermans et al., 2004). In particular, some authors suggest that sensory information may pass through both trial-and-error and declarative memory systems independently (White & McDonald, 2002) before reaching output, motor systems. Our results provide direct support to theories of multiple learning systems by demonstrating that feedback condition can be robustly classified based on functional networks. Furthermore, in line with a cooperative interaction view, we show that the output somato-motor module couples differentially with basal ganglia structures or with a visual module depending on feedback type (Figure 5B).

### Methodological Considerations

It has become increasingly clear that the insights obtained with a network-based approach are complementary and, for the most part, distinct from those obtained with univariate or multivariate activation approaches (Bassett et al., 2015; Siebenhuhner, Weiss, Coppola, Weinberger, & Bassett, 2013). Activation-based approaches typically rely on the assumption that the regions that are relevant for a cognitive process respond with different average intensities during that process in comparison to a baseline (Friston, 2005). In contrast, the assumptions in network-based approaches are that the pattern of statistical dependencies *between* regions is what varies between conditions, requiring neither an overall bulk change in average activity nor the definition of a baseline to which all conditions are compared (E. T. Bullmore & Bassett, 2011). While activation-based studies have provided us with many important and meaningful insights on behavioral and cognitive neuroscience over the course of the last two decades, network studies in task contexts have flourished more slowly and only recently the tools, statistics, and diagnostic approaches necessary for their analyses and interpretation have started to be developed (Medaglia et al., 2015). In this context, the network-based approach presented here supplements the vast activation-based literature on value learning.

Our study offers important methodological advancements for the network neuroscientist’s toolkit. First and foremost, a major contribution of our study is the direct relationship between variables derived from behavior and modulations in functional connectivity. A key advantage of this technique over more conventional neuroimaging approaches is the ability to infer the specific functional interactions in the brain that are associated with changes in behavior (Shehzad et al., 2014). Second, our work is also part of a growing set of studies that use completely data-driven analysis approaches to identify network components relevant for a cognitive process, and a cross-validated procedure for out-of-sample prediction, which may reduce the risk of overfitting and improve generalizability (Finn et al., 2015; Rosenberg et al., 2015; Shirer, Ryali, Rykhlevskaia, Menon, & Greicius, 2012; Turk-Browne, 2013).

Our study used a relatively small sample size (*N* = 16), and we therefore sought to decrease the potential for false positives by extending the study longitudinally and collecting data for each subject over four scan sessions, providing 64 scans in which to study the learning of value. We note that this approach markedly reduced the within-subject variability, which we complemented with stringent statistical testing using Bonferroni corrections for multiple comparisons. This procedure adjusts familywise error rates without requiring any assumptions about dependence among the individual tests (Goeman & Solari, 2014). While network-specific methods such as the network-based statistic (Zalesky, Fornito, & Bullmore, 2010) could have yielded a larger number of significant results for edgewise inferences across networks, the more conservative (and always valid) Bonferroni approach yielded a reasonable number of structures amenable to interpretation.

### Implications for Cognitive and Clinical Neuroscience

The learning of value is a fundamental prerequisite for healthy adult human behavior. A natural question that arises from this work is how the network architecture of value learning reconfigures as children develop from infants through adolescence and into adulthood, a process that is known to be accompanied by changes in both structural (Betzel et al., 2014) and functional (Gu et al., 2015) brain networks. Moreover, our results motivate the hypothesis that the normative characteristics of network reconfiguration that we observe in this study will be fundamentally altered in patients with deficits in reinforcement learning behavior, and in fronto-cingulate cognitive control systems, including individuals with Parkinson’s disease or schizophrenia. Finally, given recent evidence (Braun et al., 2016) it could be interesting in future studies to assess neurotransmitter-level drivers of these reconfiguration dynamics, and their dependence on cellular-level mechanisms of synaptic plasticity.

## METHODS

### Participants

Twenty human participants (nine female; ages 19–53 years; mean age = 26.7 years) with normal or corrected vision and no history of neurological disease or psychiatric disorders were recruited for this experiment. All participants volunteered and provided informed consent in writing in accordance with the guidelines of the Institutional Review Board of the University of Pennsylvania (IRB #801929). Participants had no prior experience with the stimuli or the behavioral paradigm.

### Experimental Setup and Procedure

Participants learned the monetary value of 12 novel visual stimuli in a reinforcement learning paradigm. Learning occurred over the course of four MRI scan sessions conducted on four consecutive days. A training protocol for learning of object value lasting four days has been successfully used previously (Persichetti et al., 2015), and our behavioral pilot results indicated that this period, with approximately 1,500 trials, was appropriate for most subjects to reach their individual asymptotic performances.

The novel stimuli were three-dimensional shapes generated with a custom built MATLAB toolbox (code available at http://github.com/saarela/ShapeToolbox) and rendered with RADIANCE (Ward, 1994). ShapeToolbox allows the generation of three-dimensional radial frequency patterns by modulating basis shapes, such as spheres, with an arbitrary combination of sinusoidal modulations in different frequencies, phases, amplitudes, and orientations. A large number of shapes were generated by selecting combinations of parameters at random. From this set, we selected 12 that were considered to be sufficiently distinct from one another. A different monetary value, varying from $1.00 to $12.00 in integer steps, was assigned to each shape. These values were uncorrelated with any parameter of the sinusoidal modulations, so that visual features were not informative of value.

On each trial of the experiment, participants were presented with two shapes side by side on the screen and asked to choose the shape with the higher monetary value in an effort to maximize the total amount of money in their bank. The shape values on a given trial were independently drawn from a Gaussian distribution with mean equal to the true monetary value and the standard deviation equal to $0.50. This variation in the trial-specific value of a shape was incorporated in order to ensure that participants thought about the shapes as having worth, as opposed to simply associating a number or label with each shape.

Participants completed 20 min of the main task protocol on each scan session, learning the values of the 12 shapes through feedback. The sessions were composed of three scans of 6.6 min each, starting with 16.5 s of a blank gray screen, followed by 132 experimental trials (2.75 s each), and ending with another period of 16.5 s of a blank gray screen. Stimuli were back-projected onto a screen viewed by the participant through a mirror mounted on the head coil and subtended 4° of visual angle, with 10° separating the center of the two shapes. Each presentation lasted 2.5 s (250 ms interstimulus interval) and, at any point within a trial, participants entered their responses on a four-button response pad indicating their shape selection with a leftmost or rightmost button press. Stimuli were presented in a pseudorandom sequence with every pair of shapes presented once per scan.

Feedback was provided as soon as a response was entered and lasted until the end of the stimulus presentation period. Participants were randomly assigned to two groups depending on the type of feedback received. In the relative feedback case, the selected shape was highlighted with a green or red square, indicating whether the selected shape was the most valuable of the pair or not, respectively. In the absolute feedback case, the actual value of the selected shape (with variation) was displayed in white font. Importantly, no information about the correctness of the choice was given in the absolute feedback case. Between each run, both groups received feedback about the total amount of money accumulated up to that point.

In addition to the main learning protocol, we collected fMRI data during a functional localizer, two scans of a size judgment task, and one scan of a value judgment task. The functional localizer scans consisted of 10 s blocks of faces, scenes, objects, and scrambled objects (800 ms presentation and 200 ms interstimulus interval) as participants performed a one-back task on image repetition. The size judgment task scans consisted of consecutive presentations of shapes drawn from the set and presented with a ± 10% size modulation (1,500 ms presentation and 250 ms interstimulus interval) as participants indicated whether the shape was presented in a slightly larger or smaller variation. The value judgment task scans consisted of consecutive presentations of shapes drawn from the set (1,500 ms presentation and 250 ms interstimulus interval) as participants indicated whether the shape was one of the six least or one of the six most valuable shapes. No feedback was given in any of these tasks. Data from these additional scans were not included in any of the present analyses.

Data collection and analysis were not performed blind to the conditions of the experiment.

### Subject Exclusion Criteria

Participants were excluded on the basis of head motion and task performance. From the set of 20 participants recruited for the experiment, two were excluded because of low task performance (criterion: average task accuracy at the final scan lower than 80%, Figure S1a, Mattar et al., 2018), and two were excluded because of excessive head motion (criterion: average absolute or relative motion greater than three standard deviations away from the mean, Figure S1b, c, Mattar et al., 2018). Our investigation therefore included 16 participants (eight female; ages 19–31 years; mean age = 24.1 years), of which eight remained in each feedback condition. This sample size is consistent with accepted good practices in this field (Desmond & Glover, 2002).

### MRI Data Collection

Magnetic resonance images were obtained at the Hospital of the University of Pennsylvania using a 3.0 T Siemens Trio MRI scanner equipped with a 32-channel head coil. T1-weighted structural images of the whole brain were acquired on the first scan session using a three-dimensional magnetization-prepared rapid acquisition gradient echo pulse sequence (repetition time (TR) 1,620 ms; echo time (TE) 3.09 ms; inversion time 950 ms; voxel size 1 mm × 1 mm × 1 mm; matrix size 190 × 263 × 165). A field map was also acquired at each scan session (TR 1,200 ms; TE1 4.06 ms; TE2 6.52 ms; flip angle 60; voxel size 3.4 mm × 3.4 mm × 4.0 mm; field of view 220 mm; matrix size 64 × 64 × 52) to correct geometric distortion caused by magnetic field inhomogeneity. In all experimental runs with a behavioral task, T2*-weighted images sensitive to BOLD contrasts were acquired using a slice accelerated multiband echo planar pulse sequence (TR 2,000 ms; TE 25 ms; flip angle 60; voxel size 1.5 mm × 1.5 mm × 1.5 mm; field of view 192 mm; matrix size 128 × 128 × 80). In all resting-state runs, T2*-weighted images sensitive to BOLD contrasts were acquired using a slice accelerated multiband echo planar pulse sequence (TR 500 ms; TE 30 ms; flip angle 30; voxel size 3.0 mm × 3.0 mm × 3.0 mm; field of view 192 mm; matrix size 64 × 64 × 48).

### MRI Data Preprocessing

Cortical reconstruction and volumetric segmentation of the structural data was performed with the FreeSurfer image analysis suite (Dale, Fischl, & Sereno, 1999). Boundary-based registration between structural and mean functional image was performed with FreeSurfer *bbregister* (Greve & Fischl, 2009).

Preprocessing of the resting-state fMRI data was carried out using FEAT (FMRI Expert Analysis Tool) Version 6.00, part of FSL (FMRIB’s Software Library, http://www.fmrib.ox.ac.uk/fsl). The following prestatistics processing was applied: EPI distortion correction using FUGUE (Jenkinson, 2004); motion correction using MCFLIRT (Jenkinson, Bannister, Brady, & Smith, 2002); slice-timing correction using Fourier-space time series phase-shifting; nonbrain removal using BET (Smith, 2002); grand-mean intensity normalization of the entire 4D dataset by a single multiplicative factor; and highpass temporal filtering (Gaussian-weighted least squares straight-line fitting, with sigma = 50.0 s).

Nuisance time series were voxelwise regressed from the preprocessed data. Nuisance regressors included (a) three translation (*X*, *Y*, *Z*) and three rotation (*pitch*, *yaw*, *roll*) time series derived by retrospective head motion correction (*R* = [*X*, *Y*, *Z*, *pitch*, *yaw*, *roll*]), together with expansion terms ([*R*, *R*^2^, *R*_*t*−1_, $R_{t-1}^{2}$]), for a total of 24 motion regressors (Friston, Williams, Howard, Frackowiak, & Turner, 1996); (b) the five first principal components of nonneural sources of noise, estimated by averaging signals within white matter and cerebrospinal fluid masks, obtained with FreeSurfer segmentation tools and removed using the anatomical CompCor method (aCompCor; Behzadi, Restom, Liau, & Liu, 2007); and (c) an estimate of a local source of noise, estimated by averaging signals derived from the white matter region located within a 15 mm radius from each voxel, using the ANATICOR method (Jo, Saad, Simmons, Milbury, & Cox, 2010). Global signal was not regressed out of voxel time series because of its controversial application to resting-state fMRI data (Chai, Castañón, Öngür, & Whitfield-Gabrieli, 2012; Murphy, Birn, Handwerker, Jones, & Bandettini, 2009; Saad et al., 2012). In particular, the removal of global signal in our data could mask session-to-session variability in connectivity and potentially affect accurate estimation of long-distance connections, which are a major focus of our study. We instead follow recent guidelines that suggest that removing local white matter signal and other nonneural sources are potential reasonable alternatives to global signal regression (Murphy & Fox, 2016; Power, Schlaggar, & Petersen, 2015).

### Network Construction

Network analyses of brain function began with a definition of the interacting units (nodes) and a quantification of the interactions between those units (edges). Our study follows standard practices in the field of neuroimaging and defines nodes as a collection of contiguous voxels given by an atlas or parcellation scheme, and further defines edges as the statistical dependence between the average activity in the corresponding nodes. Consistent with previous studies of task-based functional connectivity during learning, we parcellated the brain into 112 cortical and subcortical regions, separated by hemisphere using the structural Harvard-Oxford atlas of the FMRIB (Oxford Centre for Functional Magnetic Resonance Imaging of the Brain) Software Library (FSL; Version 5.0.4). We warped the MNI152 regions into subject-specific native space using FSL FNIRT (nonlinear normalization) and nearest neighbor interpolation and calculated the average BOLD signal across all gray matter voxels within each region. The participant’s gray matter voxels were defined using the anatomical segmentation provided by FreeSurfer, projected into subject’s EPI space with *bbregister*.

We then calculated the edge weights connecting nodes by measuring the wavelet coherence between the activities of the corresponding regions. Wavelet decompositions of fMRI time series have been applied extensively to fMRI data (E. Bullmore et al., 2003, 2004), where they sensitively detect small signal changes in nonstationary time series with noisy backgrounds (Brammer, 1998). We first extracted the wavelet coefficients of the average signal within each region using the WMTSA Wavelet Toolkit for MATLAB (Percival & Walden, 2006). Given our sampling frequency of 2 s, we used scale 2 coefficients (corresponding approximately to 0.06–−0.125 Hz) to calculate the magnitude squared coherence, using the MATLAB function *mscohere*. We repeated this procedure for all pairs of regions, forming an adjacency matrix **A** for each subject and for each scan.

We note that task-related events were not regressed out or explicitly modeled in our analyses. The effects of a task with intertrial intervals as short as ours (2.75 s) on measures of functional connectivity almost completely disappear when filtered through the low-pass hemodynamic response function. Approaches that account for context-dependent psychophysiological interactions (or its generalized form; McLaren, Ries, Xu, & Johnson, 2012) are, therefore, ineffective in our data. At any rate, the removal of these effects is controversial in the network neuroscience literature, with researchers using either approach depending on specific details of the experimental design and tested hypotheses.

### Multislice Community Detection

Approaches to data clustering in networks are generally based on community detection techniques (Fortunato, 2010; Porter, Onnela, & Mucha, 2009). Here we use a generalized Louvain method (Blondel, Guillaume, Lambiotte, & Lefebvre, 2008) for optimizing modularity (Newman, 2006) developed specifically for community detection in multislice systems (Mucha, Richardson, Macon, Porter, & Onnela, 2010): systems in which multiple networks linked by an ordered or categorical dimension (time in our case) are to be examined at once. We implemented a categorical multislice modularity maximization (Jutla, Jeub, & Mucha, 2011; Mucha et al., 2010) that considers the multiple adjacency matrices as slices of a single network, enforcing consistency in node identity across slices by adding interslice connections between each node and itself across adjacent slices of the network. We then optimize the multislice modularity quality function 1, which uses the relative densities of intracommunity connections versus intercommunity connections to identify a partition of network nodes into communities or modules (Mucha et al., 2010), defined as

$$Q_{\text{multislice}}=\frac{1}{2\mu }\underset{ijsr}{\sum }\left[\left(A_{ijs}-\gamma_{s}V_{ijs}\right)\delta_{sr}+\delta_{ij}\omega_{jsr}\right]\delta (g_{is},g_{jr}),$$ (1)

where the adjacency matrix of slice *s* has components *A*_*ijs*_, the element *V*_*ijs*_ gives the components of the corresponding matrix for a null model, *γ*_*s*_ is the structural resolution parameter of slice *s*, the quantity *g*_*is*_ gives the community (i.e., “module”) assignment of node *i* in slice *s*, the quantity *g*_*jr*_ gives the community assignment of node *j* in slice *r*, the parameter *ω*_*jsr*_ is the connection strength between node *j* in slice *s* and node *j* in slice *r*, the total edge weight in the network is $\mu =\frac{1}{2}\sum_{jr}\kappa_{jr}$, the strength of node *j* in slice *s* is *κ*_*js*_ = *k*_*js*_ + *c*_*js*_, the intraslice strength of node *j* in slice *s* is *k*_*j*_*s*, and the interslice strength of node *j* in slice *s* is $c_{js}=\sum_{r}\omega_{jsr}$. We employ the Newman-Girvan null model within each layer by using

$$V_{ijs}=\frac{k_{is}k_{js}}{2m_{s}},$$ (2)

where $m_{s}=\frac{1}{2}\sum_{ij}A_{ijs}$ is the total edge weight in slice *s*. The free-parameters are the structural resolution parameters, *γ*_*s*_, and the interslice coupling parameters, *ω*_*jsr*_, here assumed to be constant (*γ*_*s*_ = *γ*, ∀*s* and *ω*_*jsr*_ = *ω*, ∀*j* and ∀*s* ≠ *r*, meaning that node *j* in slice *s* connects to node *j* in every slice *r* ≠ *s* with weight *ω*). These parameters control the size of communities within a given layer and the number of communities discovered across layers, respectively.

In order to obtain a single, representative, partition of the brain into network communities, we performed 100 optimizations of the modularity function for each participant, using nonoverlapping 60-s windows and the standard parameters of *γ* = 1.0 and *ω* = 1.0. We then calculated the module allegiance matrix (Mattar, Cole, Thompson-Schill, & Bassett, 2015), a 112 × 112 matrix whose *i*, *j* elements correspond to the probability that regions *i* and *j* belong to the same community across all optimizations, scans, and participants. By repeating the procedure of maximizing the modularity function on the module allegiance matrix 100 times and recalculating a new module allegiance matrix, a consensus partition is considered to have been obtained when all 100 optimizations of the modularity function are identical. The structural resolution parameter used in the optimizations of the single-layer module allegiance matrix can be tuned to yield a different level of the hierarchical organization of the network. We chose a value of *γ* = 1.4, which yielded the maximum number of communities present simultaneously in both hemispheres of the brain.

## AUTHOR CONTRIBUTIONS

Marcelo G. Mattar: Conceptualization; Formal analysis; Investigation; Methodology; Project administration; Software; Visualization; Writing – original draft. Sharon L. Thompson-Schill: Conceptualization; Funding acquisition; Project administration; Supervision; Writing – original draft. Danielle S. Bassett: Conceptualization; Methodology; Project administration; Resources; Writing – original draft.

## FUNDING INFORMATION

We acknowledge support from the John D. and Catherine T. MacArthur Foundation (DSB), the Alfred P. Sloan Foundation (DSB), the Army Research Laboratory, and the Army Research Office through contract numbers W911NF-10-2-0022 (DSB) and W911NF-14-1-0679 (DSB), the National Institute of Health (2-R01-DC-009209-11 (Thompson-Schill), 1R01HD086888-01 (DSB), R01-MH107235 (Gur), R01-MH107703 (Satterthwaite), R01MH109520 (Cole), 1R01NS099348 (DSB;Litt), and R21-M MH-106799 (DSB;Satterthwaite)), the Office of Naval Research (DSB), and the National Science Foundation (BCS-1441502, CAREER PHY-1554488, BCS-1631550, and CNS-1626008 all to DSB). The content is solely the responsibility of the authors and does not necessarily represent the official views of any of the funding agencies.

## TECHNICAL TERMS

Visual network: A set of brain regions, primarily located throughout the occipital and temporal lobes, involved in the representation of visual objects and their form.
Valuation network: A set of brain regions involved in the representation of value information—a useful signal for decision-making—spanning the basal ganglia, anterior cingulate, ventro-medial prefrontal, and orbito-frontal cortices.
Functional network: A set of distributed brain regions that display coherent activity with one another over the course of a certain temporal extent.
Bonferroni correction: A correction applied to a statistical test with the goal of adjusting the false-positive rate when performing multiple hypothesis tests. The correction amounts to dividing the threshold under which the null hypothesis is rejected by a factor corresponding to the number of tests conducted.
Consensus partition: A partition of a network that is representative of a set of other (potentially distinct) partitions. Here, we define a consensus partition as groups of nodes that are consistently assigned to the same community across participants and across time.
Community structure: A partition of a network into groups of nodes (overlapping or not) that are densely connected, with sparser connections between groups.
Leave-one-out cross-validation: A model evaluation method where a predictive model is derived based on all the data except for one point (e.g., one subject), which is then used to make a prediction for the remaining point.
Cross-validation fold: The subset of the data used to derive a predictive model.
Permutation test: A statistical test of significance where the distribution of the test statistics under the null hypothesis is generated by permuting the labels of the data points.
