# Supplementary material for: The network architecture of value learning
Source: Netw Neurosci. 2018 Jun 1;2(2):128–49. doi: 10.1162/netn_a_00021 (PMC6130435; doi:10.1162/netn_a_00021)
Supplement: Supplementary file 1 [file netn-02-128-s001.pdf]

Mattar, M. G., Thompson-Schill, S. L., & Bassett, D. S. (2017). Supplementary Material for
"The network architecture of value learning." Network Neuroscience, 2(2), 128-149.
https://doi.org/10.1162/netn_a_00021

The network architecture of value learning

Supplementary Material

Marcelo G. Mattar[1,2], Sharon L. Thompson-Schill[1] and Danielle S. Bassett[*2,3]

[1]Department of Psychology, University of Pennsylvania, Philadelphia, PA 19104, USA

[2]Department of Bioengineering, University of Pennsylvania, Philadelphia, PA 19104, USA

[3]Department of Electrical & Systems Engineering, University of Pennsylvania, Philadelphia, PA 19104, USA

[*]Corresponding author: dsb@seas.upenn.edu

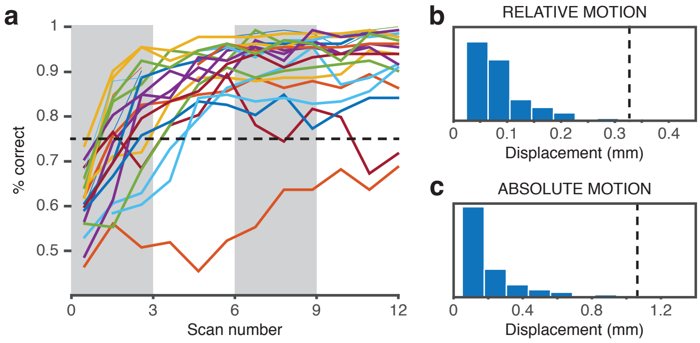

Figure S1: *Related to Figure 1.* **Subject exclusion criteria.**
(a) Individual learning curves for each of the 20 participants. Participants with task performance below 75% at the last run were excluded from the analyses in the main text.
(b) Average relative motion within scan runs. Participants with average relative motion larger than three standard deviations away from the mean in two of more scans were excluded from the analyses in the main text.
(c) Average absolute motion within scan runs. Participants with average relative motion larger than three standard deviations away from the mean in two of more scans were excluded from the analyses in the main text.

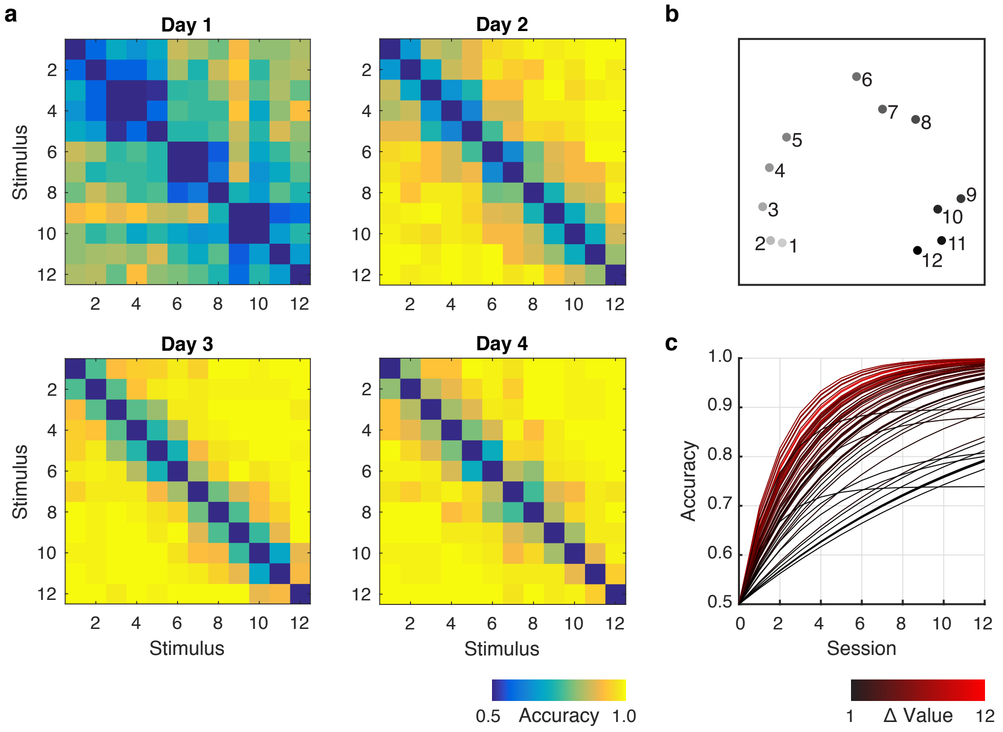

Figure S2: *Related to Figure 1.* **Discriminability of individual stimulus pairs.**
(a) Average accuracy on each day in selecting the most valuable shape from the pair. Learning progressed as expected, with pairs of stimuli with similar value having worse task accuracy. Notice that all pairs of stimuli could be sufficiently discriminated as evidenced by accuracy values over 0.75 and by the absence of any pair with lower accuracy.
(b) Multidimensional scaling analysis. The two dimensions accounting for the most variance (accounting for 61% when combined) are displayed. In this plot, stimulus similarity can be inferred by the proximity in the reconstructed space. These results show that stimuli were roughly organized in a circular fashion, with proximity clearly reflecting value similarity. The circular arrangement reflects the fact that accuracy is at ceiling for all pairs of stimuli whose value distance is beyond a certain threshold (approximately $5).
(c) Individual learning curves for each pair of stimuli. We fit each curve with an exponential curve with two free-parameters: the exponential parameter and the asymptotic accuracy. All fits were constrained to start at chance level. We observed accuracy for all but a few pairs increased steadily over the course of the experiment towards a maximum level of 1.0. The only major exception was the pair ($5, $6) whose accuracy converged to 0.74, which, as seen on Fig. 1a, are easily distinguishable based on visual similarity alone.

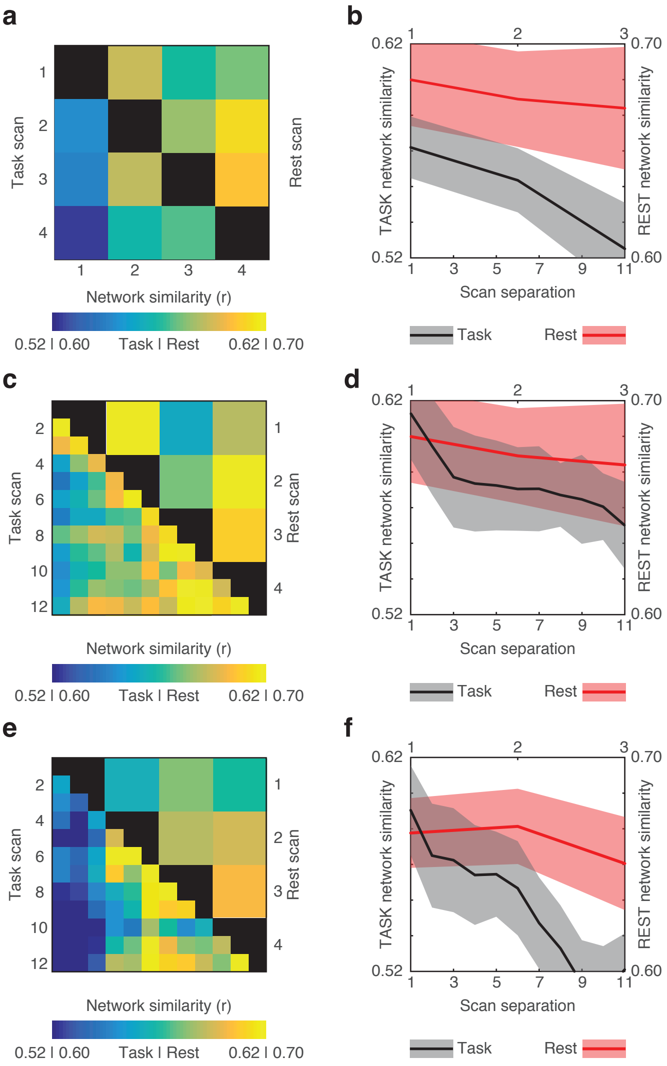

Figure S3: *Related to Figure 2.* **Network evolution throughout learning.**
(a) Network similarity limited to first task scans on each day.
(b) Corresponding average network similarity as a function of temporal separation between corresponding scans. Black line: task scans; Red line: rest scans.
(c) Network similarity for participants from *absolute* feedback group.
(d) Corresponding average network similarity as a function of temporal separation between corresponding scans. Black line: task scans; Red line: rest scans.
(e) Network similarity for participants from *relative* feedback group.
(f) Corresponding average network similarity as a function of temporal separation between corresponding scans. Black line: task scans; Red line: rest scans.

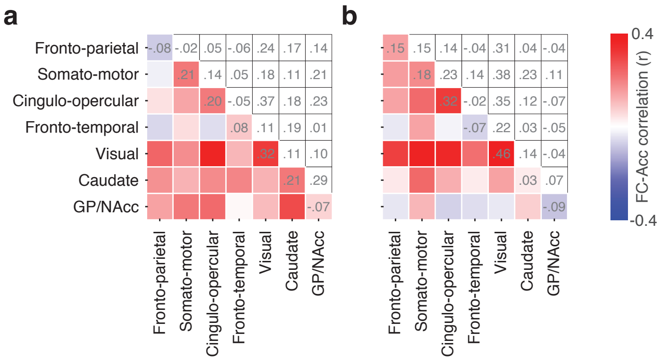

Figure S4: *Related to Figure 3.* **Community-level interactions related to value learning**
(a) Correlation between average edge weight within/between communities and task accuracy for *absolute* feedback group.
(b) Correlation between average edge weight within/between communities and task accuracy for *relative* feedback group.

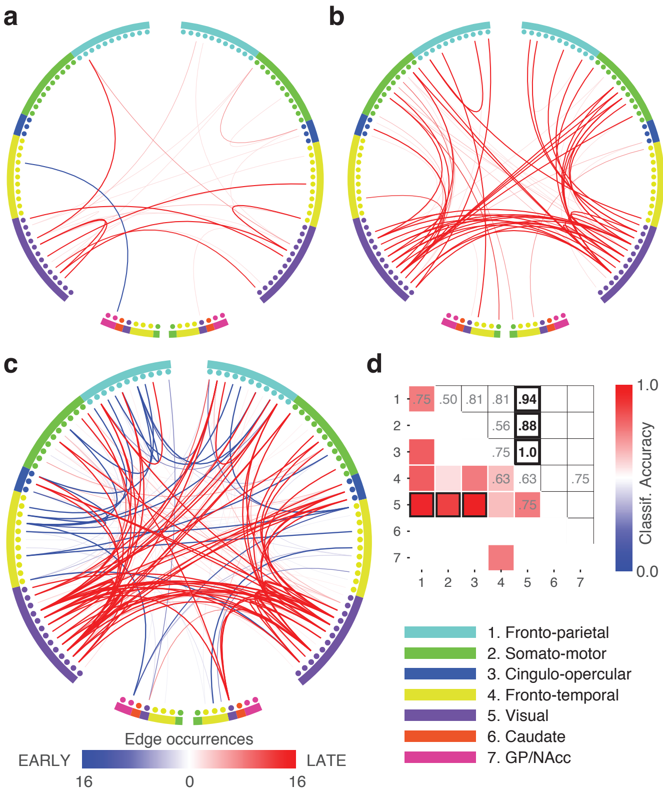

Figure S5: *Related to Figure 4.* **Prediction of learning stage from functional networks**
(a) Related to Fig. 4a but limited to *absolute* feedback group. Using a cross-validation procedure, the subset of edges where a one-sample t-test yielded a test statistic with P-value lower than 0.001 was selected as a *predictive network*. The figure shows the number of cross-validation folds in which each edge was identified as part of the predictive network. Edges whose strength correlated positively with task accuracy are displayed in red. Edges whose strength correlated negatively with task accuracy are displayed in blue.
(b) Related to Fig. 4a but limited to *relative* feedback group.
(c) Related to Fig. 4a (data from both groups), but forming predictive networks after the removal of the average network strength at each scan. Using this alternative cross-validation procedure, the number of edges present in the predictive networks ranged from 105 to 154 ($M = 132.5, SD = 13.7$) and classification was correct in all 16 participants (accuracy: 100% vs. chance: 50%; one-tailed binomial test: $P = 1.525 \times 10^{-5}$).
(d) Related to Fig. 4b (data from both groups), but forming predictive networks after the removal of the average network strength at each scan. Cell colors and numbers represent classification accuracy in labeling held-out data as coming from scans early or late in the learning process. Data from the left-out participant was significantly classified above chance (50%) when the predictive network was comprised of edges connecting: (i) visual and fronto-parietal modules (accuracy: 93.75%; one-tailed binomial test, adjusted P-value: $P = 0.0054$); (ii) visual and somato-motor modules (accuracy: 87.50%; one-tailed binomial test, adjusted P-value: $P = 0.044$); and (iii) visual and cingulo-opercular modules (accuracy: 100%; one-tailed binomial test, adjusted P-value: $P = 0.00032$).

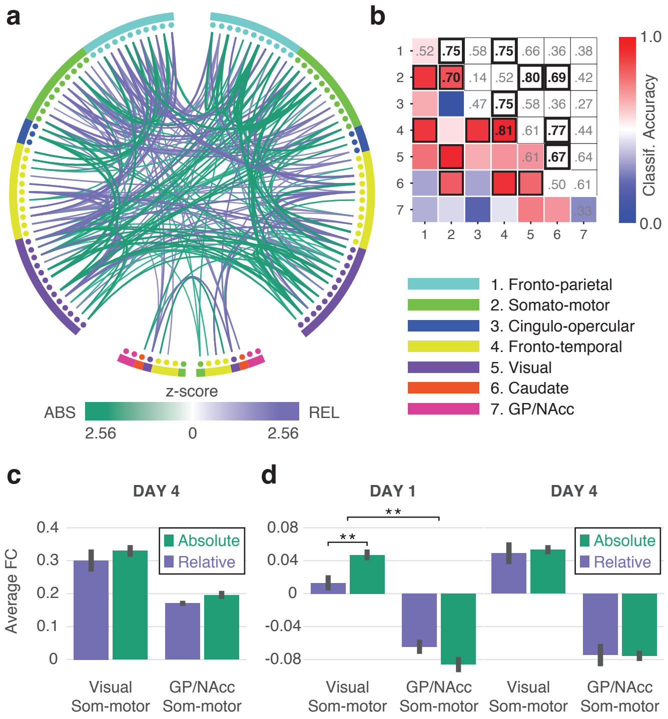

Figure S6: *Related to Figure 6.* **Prediction of feedback-type from functional networks**
(a) We used a support-vector machine with leave-two-out cross-validation to classify feedback type based on the entire set of edge weights. On each cross-validation fold, the w-map represents how useful each feature (edge weight) is at discriminating between conditions. The figure displays the average z-scored w-map, limited to connections with $z > 1.96$ (green: absolute feedback; purple: relative feedback). Notice the similarity with Fig. 5a.
(b) To gain insight into the specific modules that enable classification of feedback-type, we conducted the analyses in the main text separately for each pair of communities, selecting the top 10% edges at each cross-validation fold. Cell colors and numbers represent classification accuracy in labeling held-out data as coming from participants in the absolute-vs.-relative feedback group. Communities whose interactions classified held-out data significantly (permutation tests, Bonferroni corrected at $\alpha = 0.05$) are highlighted. Community order is displayed in the bottom-right.We observed that interactions involving somato-motor, fronto-temporal, and caudate modules were modulated by feedback type.
(c) Related to Fig. 5b. Average functional connectivity on **DAY 4** between Somato-motor and Visual, and between Somato-motor and GP/NAcc modules displayed separately for each feedback group. Neither difference nor the interaction was significant (two-way ANOVA interaction: $F(1, 28) = 2.13, P = 0.88$; two-sample t-tests: $t(14) = 0.86, P = 0.40$, $t(14) = 1.82, P = 0.09$).
(d) Related to Fig. 5b. Average functional connectivity on **DAY 1** (left) and **DAY 4** (right) between Somato-motor and Visual, and between Somato-motor and GP/NAcc modules displayed separately for each feedback group, after subtracting the mean connectivity from each adjacency matrix. In line with our hypotheses, we observed a significant interaction (two-way ANOVA interaction: $F(1, 28) = 10.8, P = 0.0027$), with connectivity between somato-motor and visual modules being stronger for the *absolute* feedback group (two-sample t-test on Fisher normalized correlation values: $t(14) = 3.02, P = 0.0092$), and connectivity between the somato-motor and basal ganglia modules being stronger (though not significantly) for the *relative* feedback group (two-sample t-test on Fisher normalized correlation values: $t(14) = 1.72, P = 0.11$). The interaction and differences were not significant on day 4 (two-sample t-tests: $t(14) = 0.30, P = 0.77$, $t(14) = 0.07, P = 0.95$; two-way ANOVA interaction: $F(1, 28) = 0.068, P = 0.80$).

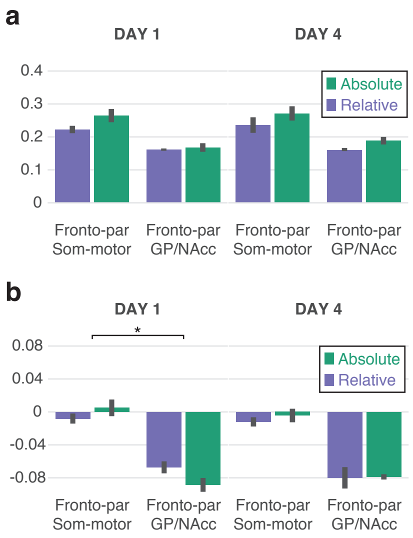

Figure S7: *Related to Figure 6.* **Prediction of feedback-type from functional networks**
(a) Related to Fig. 5b. Average functional connectivity on **DAY 1** (left) and **DAY 4** (right) between fronto-parietal and somato-motor, and between fronto-parietal and GP/NAcc modules displayed separately for each feedback group. The interaction and differences were not significant on day 1 (two-sample t-tests: $t(14) = 1.95, P = 0.071$, $t(14) = 0.49, P = 0.63$; two-way ANOVA interaction: $F(1, 28) = 2.06, P = 0.16$) nor on day 4 (two-sample t-tests: $t(14) = 1.17, P = 0.26$, $t(14) = 2.25, P = 0.041$; two-way ANOVA interaction: $F(1, 28) = 0.065, P = 0.80$).
(b) Related to Fig. 5b. Average functional connectivity on **DAY 1** (left) and **DAY 4** (right) between fronto-parietal and somato-motor, and between fronto-parietal and GP/NAcc modules displayed separately for each feedback group, after subtracting the mean connectivity from each adjacency matrix. We observed a significant interaction (two-way ANOVA interaction: $F(1, 28) = 4.51, P = 0.043$), with connectivity between fronto-parietal and somato-motor modules being stronger (though not significantly) for the *absolute* feedback group (two-sample t-test on Fisher normalized correlation values: $t(14) = 1.10, P = 0.29$), and connectivity between the fronto-parietal and basal ganglia modules being stronger (though not significantly) for the *relative* feedback group (two-sample t-test on Fisher normalized correlation values: $t(14) = 1.93, P = 0.074$). The interaction and differences were not significant on day 4 (two-sample t-tests: $t(14) = 0.78, P = 0.45$, $t(14) = 0.079, P = 0.94$; two-way ANOVA interaction: $F(1, 28) = 0.16, P = 0.69$).

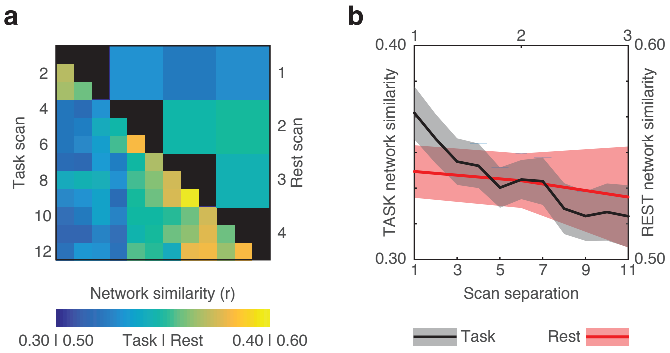

Figure S8: *Related to Figure 2.* **Network similarity after global signal regression.**
(a) Lower diagonal: Network similarity (Pearson correlation coefficient) between functional connectivity matrices corresponding to each pair of task scans. Upper diagonal: Network similarity (Pearson correlation coefficient) between functional connectivity matrices corresponding to each pair of rest scans, conducted over the same period of time as the task scans ($N = 16$).
(b) Average network similarity as a function of temporal separation between corresponding scans. Black line: task scans; Red line: resting-state scans ($N = 16$).

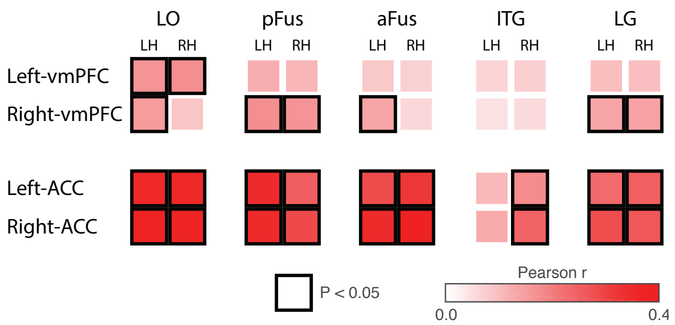

Figure S9: **Correlation between weight changes on specific network edges and task accuracy** We examined the heterogeneity of effects across regions of the visual cortex in order to determine the level of granularity across regions involved in object perception and nearby regions. We considered five visual areas (in both hemispheres): Lateral Occipital Cortex (LO), a region known to be involved in object perception, and a region immediately anterior and ventral, Inferior Temporal Gyrus (ITG); A second region involved in object perception, Posterior Fusiform Gyrus (pFus), its anterior counterpart in the Temporal Occipital Fusiform Cortex (aFus), and the region immediately medial to it, the Lingual Gyrus (LG). We also considered two regions in the value network (in both hemispheres): the ventral-medial Prefrontal Cortex (vmPFC) and the Anterior Cingulate Cortex (ACC). We then calculated, for each subject, the Pearson correlation between edge weight and learning rate. Our results show an overall trend for positive correlation values, indicating that links between regions of the visual and value networks tend to grow stronger as learning progresses. Yet, this pattern was not equally significantly expressed in all regions of the visual cortex. In particular, edges connecting ITG with vmPFC; left-ITG with ACC; and LG with left-vmPFC were not significantly correlated with learning rate. These results suggest that learning requires changes in network edges that are relatively spatially specific.

Insights into fundamental constraints on dynamic network architecture

In the main text, we describe specific network components at various levels that change in concert with the learning of value. Our initial results demonstrate that functional networks, in general, change gradually over time as subjects learn stimulus values (Fig. 2). In subsequent sections, we then examined the role of specific components of the network at the scales of nodes and edges (Fig. 3; Fig.4; Fig. 5). In the present section, we investigate the question of how these components relate to the mesoscale architecture of the network, in order to gain a greater insight into the properties that may relate to the ability of a network component to modulate (with) learning. In particular, we focus on the analysis of node flexibility, which has been used successfully in the past to describe the reconfiguration patterns of network modules throughout learning. Specifically, network flexibility examines the degree to which each brain region changes its allegiances to network modules over time [1]. By grouping regions according to whether they are more or less flexible than a suitable statistical null-model, the temporal core and periphery of the network can be reliably identified.

In the context of a motor-skill learning task, the core-periphery organization of a network has been used to understand how putative functional modules are linked. Specifically, the core is composed of regions whose connectivity change little over time (sensorimotor and visual regions in the case of a motor-skill learning task), while the periphery is composed of regions whose connectivity changes frequently (primarily multimodal association regions), and the separation between these two large groups is predictive of learning rate in a motor task [1]. In a subsequent study, regions of the network core were also observed to have consistent module allegiance over time, again suggesting that the strength of connections within modules of the core is high and stable, and that the connections that change most notably with learning are the ones connecting different modules of the network core [2]. These results have been understood in terms of domain-specific *versus* domain-general computations. In particular, regions that support computations specific for a particular task are more likely to robustly combine with other regions that support similar computations. In contrast, regions that support very generic computations can connect with a diverse set of regions that, presumably, are recruited across a wide range of tasks [3]. Based on the existing empirical and theoretical evidence, we posited that learning primarily modulates functional connectivity between regions of the temporal core of a dynamic network. In other words, we hypothesized that core regions consistently recruited for the execution of the task, while retaining their module affiliation over time, would reconfigure slowly at the timescale of learning. In contrast, periphery regions reflecting domain-general computations should exhibit more frequent and more inconsistent module affiliation and, in turn, not change their connections robustly in concert with learning.

To test these hypotheses, we first examined the temporal variability of community structure by com-

puting the *flexibility* f_i of each region i [1]. To calculate flexibility, we identified the dynamic community structure over the 4 days of learning based on multilayer representations of temporal networks in 60 second windows [4]. We then calculated the flexibility of each region as the relative frequency with which it changed its allegiances to network communities over time: a high value of flexibility indicates that a region changes community affiliation frequently. We assessed the relationship between network flexibility and learning by calculating the amount of variance (R-squared) in task accuracy explained by each region: that is, the average R-squared over all edges departing from that region. We observed a strong negative relationship between node flexibility and variance explained: regions with low flexibility explained larger amounts of variance in task accuracy than regions with high flexibility (Pearson's $r = -0.49, t(15) = -5.37, P < 0.0001$; Fig. S10a).

We wished to test whether this relationship could be accounted for by a higher flexibility in nodes with lower signal-to-noise ratio. To that end, we calculated, for each node, the temporal SNR (tSNR), defined as the mean signal of the fMRI time series divided by its standard deviation across time [5]. We then averaged this quantity across scan sessions, obtaining an estimate of tSNR for each region and each subject. We observed node flexibility and node tSNR were not significantly correlated (Pearson's $r = -0.12; t(15) = -0.34, P = 0.74$). In addition, we tested whether the negative relationship between node flexibility and variance explained would be maintained after controlling for node tSNR. To that end, we regressed out the tSNR value of each node from its flexibility value and recomputed the relationship between node flexibility and variance explained. We observed that the relationship remained negative and highly significant (Pearson's $r = -0.50, t(15) = -4.36, P = 0.00056$).

These results suggest that the regions whose functional connectivity tracks task accuracy are those in a temporal core of relatively rigid areas whose affiliation with functional modules remains steady throughout task practice [1]. To determine whether this is indeed the case, we next categorized brain regions into *temporal core* and *temporal periphery* by assessing whether a region's flexibility was less than or greater than expected in a nodal null model, respectively [1]. Using this approach, we observed that the network core – dynamically rigid regions with dense connectivity – encompasses regions in the visual, frontal (specifically, the ventral-medial and fronto-polar regions), and right motor areas. In contrast, the network periphery – flexible regions with weak connectivity – encompasses regions in the anterior temporal lobe and subcortical structures (Fig. S10b). Moreover, the communities that we previously observed to be related to learning were not only the most rigid ones (Fig. S10a,b), but were also less flexible than expected by their size alone (Fig. S10c).

While it may seem contradictory that regions of the core are simultaneously the most rigid and the most involved in learning, we note that the rigidity corresponding to low flexibility simply indicates that the

region does not change module affiliation very frequently – indeed, a region may certainly change module affiliation, for example, over the course of days, and still be classified as part of the core. Relatedly, a region that changes its module affiliation very frequently is unlikely to be involved in the gradual learning that occurs over the course of many days, and is instead more likely to reflect domain-general processes, related to or not related to the task. These points support our theoretical reasoning that core regions, which have stable partners during task execution, are also the ones more likely to change with learning.

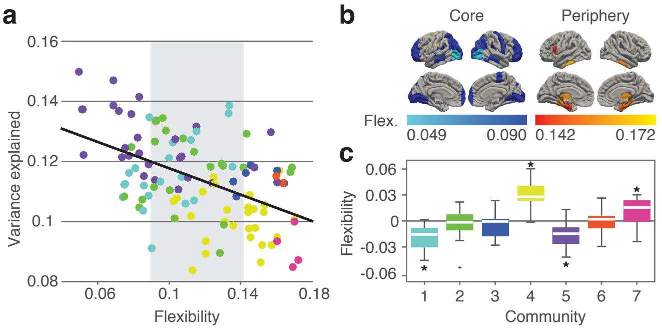

Figure S10: **Regions in the network core are more associated with learning than regions in the network periphery.** *(a)* The variance in task accuracy explained by each of the 112 nodes (calculated as the average variance explained across all edges departing from a node) is negatively correlated with node flexibility (Pearson's $r = -0.49, P < 0.001$). Each circle corresponds to a brain region and is colored with the color of its corresponding community (Fig. 3d). The shaded area corresponds to the 95% confidence interval of the nodal null model described in (b), which separates nodes into a temporal core and a temporal periphery. *(b)* A *nodal null model* was constructed by rewiring the ends of the multilayer network's inter-layer edges uniformly at random 100 times. The temporal "core" was then defined as the set of regions whose mean nodal flexibility was below the 2.5% confidence bound of the null-model distribution, and, similarly, the temporal "periphery" was defined as the set of regions whose mean nodal flexibility was above the 97.5% confidence bound of the null-model distribution. The temporal core consists of regions of the visual, frontal, and (right) motor cortices. The temporal periphery consists of subcortical regions and regions of the anterior temporal lobe. *(c)* Average flexibility within each network community controlling for community size. Two communities exhibited flexibility significantly below that expected by its size: (i) fronto-parietal ($f = -0.019, P < 0.001$), and (ii) visual ($f = -0.015, P < 0.001$); and two communities exhibited flexibility significantly above that expected by its size: (i) fronto-temporal ($f = 0.032, P < 0.001$), and (ii) GP/NAcc ($f = 0.011, P = 0.019$).

References

[1] D. S. Bassett, N. F. Wymbs, M. P. Rombach, M. A. Porter, P. J. Mucha, and S. T. Grafton, "Task-based core-periphery organization of human brain dynamics," *PLoS Comput Biol*, vol. 9, no. 9, p. e1003171, 2013.

[2] D. S. Bassett, M. Yang, N. F. Wymbs, and S. T. Grafton, "Learning-induced autonomy of sensorimotor systems," *Nature neuroscience*, vol. 18, no. 5, pp. 744–751, 2015.

[3] E. Fedorenko, "The role of domain-general cognitive control in language comprehension," *Frontiers in psychology*, vol. 5, p. 335, 2014.

[4] P. J. Mucha, T. Richardson, K. Macon, M. A. Porter, and J.-P. Onnela, "Community structure in time-dependent, multiscale, and multiplex networks," *science*, vol. 328, no. 5980, pp. 876–878, 2010.

[5] M. Welvaert and Y. Rosseel, "On the definition of signal-to-noise ratio and contrast-to-noise ratio for fmri data," *PloS one*, vol. 8, no. 11, p. e77089, 2013.
